# Tumor microenvironment and immunotherapy of oral cancer

**DOI:** 10.1186/s40001-022-00835-4

**Published:** 2022-10-08

**Authors:** Chang Liu, Min Wang, Haiyang Zhang, Chunyan Li, Tianshou Zhang, Hong Liu, Song Zhu, Jie Chen

**Affiliations:** 1 Hospital of Stomatology, Jilin University, Changchun, 130021 People’s Republic of China; 2 Key Laboratory of Polymer Ecomaterials, Changchun Institute of Applied Chemistry, Chinese Academy of Sciences, Changchun, 130022 People’s Republic of China

**Keywords:** Oral cancer, Immunotherapy, Tumor microenvironment, Immune checkpoint blockade, Immune tolerance

## Abstract

Oral cancer is one of the most common malignant tumors of the head and neck, not only affects the appearance, but also affects eating and even endangers life. The clinical treatments of oral cancer mainly include surgery, radiotherapy, and chemotherapy. However, unsatisfactory therapeutic effect and toxic side effects are still the main problems in clinical treatment. Tumor microenvironment (TME) is not only closely related to the occurrence, growth, and metastasis of tumor but also works in the diagnosis, prevention, and treatment of tumor and prognosis. Future studies should continue to investigate the relationship of TME and oral cancer therapy. This purpose of this review was to analyze the characteristics of oral cancer microenvironment, summarize the traditional oral cancer therapy and immunotherapy strategies, and finally prospect the development prospects of oral cancer immunotherapy. Immunotherapy targeting tumor microenvironment is expected to provide a new strategy for clinical treatment of oral cancer.

## Introduction

Oral cancer is one of the most common malignant tumors of the head and neck [1]. Most of them are squamous cell carcinoma (OSCC), which is associated with mucosal variation [2]. Oral cancer usually occurs at the bottom of the mouth, lips, tongue, gums, and the inside of the cheek, including soft or hard palate cancer, gingival cancer, oropharyngeal cancer, lip cancer, tongue cancer, jaw bone cancer, salivary gland cancer, oral base cancer, facial skin mucosa, and maxillary sinus cancer [3]. Oral cancer can be caused by many factors, including long-term alcoholism, poor oral hygiene, excessive exposure to sunlight, betel nut, long-term foreign body stimulation, malnutrition, mucosal leukoplakia or erythema, and oral ulcers. Oral cancer not only affects the appearance but also affects eating and even endangers life. There are many treatment methods for oral cancer [4, 5]. Currently, the conventional treatment methods are surgery therapy, radiotherapy, and chemotherapy [6]. Surgery is generally based on the size of the tumor to choose the appropriate range of resection; some tongue cancer patients may be seriously affected in eating and speaking after surgery [7]. Radiation therapy belongs to local treatment. During radiotherapy, local skin and mucous membrane may be damaged, including radioactive skin injury, radioactive mucous membrane injury, and radioactive stomatitis [8]. Chemotherapy belongs to systemic treatment, which may lead to pancytopenia and severe nausea gastrointestinal reaction [9]. Traditional Chinese Medicine treatment is mainly used for the treatment of oral cancer complications caused by other strategies [10]. Nevertheless, there are few safe and efficient clinical treatment strategies for oral cancer, which is mainly due the complex tumor microenvironment (TME) [11]. TME is closely related to the occurrence, metastasis, and recurrence of malignant tumors and is composed of non-cellular components in the extracellular matrix (ECM) and cellular components, such as fibroblasts and immune cells [12]. TME performs signal transmission through autocrine–paracrine signaling pathway, thus controlling the proliferation and metastasis of tumor cells [13]. In 1863, Virchow et al. first proposed the concept of tumor microenvironment, pointing out the relationship between inflammation and cancer [14]. With the rapid development of tumor cytology and other disciplines, studies on the mechanism of tumor genesis, development, and metastasis have been continuously deepened [15, 16]. Abnormal TME could promote the invasion and metastasis of tumors, as well as hindering effective drug diffusion and immune cell invasion [17, 18]. In the early stage, tumor microenvironment was able to restrain tumor growth and then the TME gradually transformed into the "soil" to assist the survival and development of malignant tumor [19]. Under its influence, tumor microenvironment is characterized by slight acidity, hypoxia, high reactive oxygen species (ROS), high osmotic pressure, and abnormal vasculature [20, 21]. In addition, tumor microenvironment inhibited the proliferation and activity of tumor-specific T cells (CTLs) and promoted regulatory T cells (Tregs), resulting in a decrease in the secretion of immune-activating cytokines, such as tumor necrosis factor (TNF-α) and interferon γ (IFN-γ), and an increase in the secretion of immunosuppressive cytokines, such as transforming growth factor (TGF-β) and interleukin-10 (IL-10). TME could also assist tumor cells to escape immune surveillance, hinder dendritic cells (DCs) from presenting antigens, inhibit the activity of CTLs, and ultimately achieve immune tolerance and immune escape of tumor cells [22]. In recent years, with the deepening understanding of the role of TME in tumor development, immunotherapy based on TME shows an important research value and clinical significance.

There are complex network relationships among the components of TME, and a deep understanding of the composition and characteristics of TME is the premise of developing immunotherapy based on the tumor microenvironment. In this review, we highlighted the tumor microenvironment and immunotherapy strategies of oral cancer. First, the compositions and functions of TME in oral cancer were presented and reviewed. More importantly, the recent development of treatment strategies for oral cancer was summarized, especially for immunotherapy. Finally, the promising development directions of immunotherapy for oral cancer in the future were prospected.

## Microenvironment of oral cancer

**Fig. 1:**
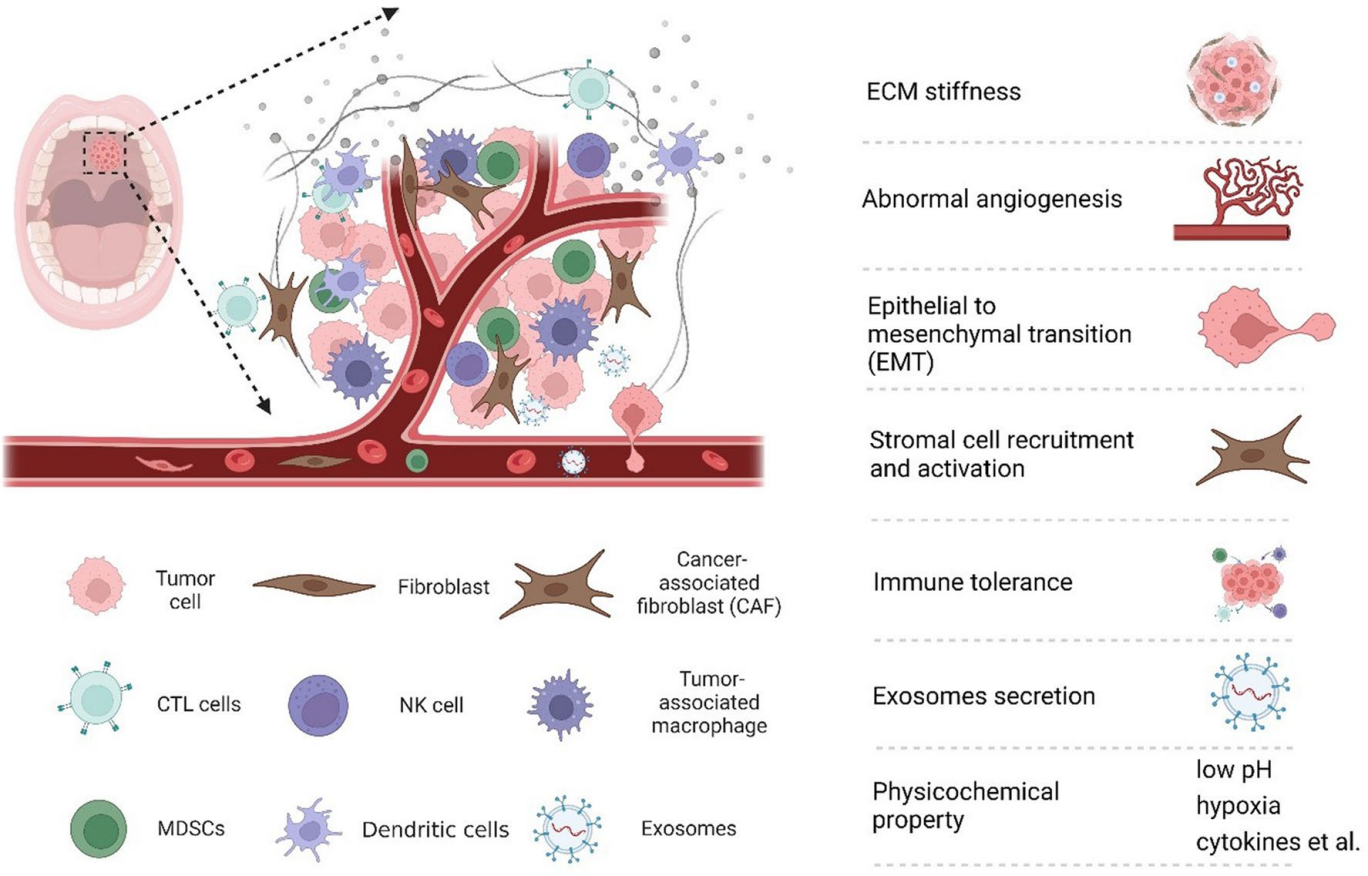
Schematic diagram of the microenvironment of oral cancer

The occurrence, development, and metastasis of tumors are closely related to external environment of oral cancer cells, including the structure, function, and metabolism of tumor tissue, as well as the internal environment of cancer cells (Fig. 1). Tumors and their microenvironment are interdependent and antagonistic [23].

## Tumor extracellular matrix

ECM is a complex network structure composed of macromolecules secreted by tumor cells into the extracellular stroma [24]. The regulatory abnormalities of ECM are a prominent feature of TME. In the process of tumor genesis and development, tumor cells promote the formation of ECM; in turn, ECM can also regulate the related proliferation of tumor cells. The interaction between tumor cells and ECM can activate multiple specific signaling pathways [25, 26]. Therefore, an adequate understanding of ECM dysregulation is beneficial to identify potential tumor therapeutic targets. The structure and function of collagen, fibronectin, elastin, and other ECM components will be discussed.

Collagens accounts for about 90% of human external matrix and 30% of total protein [27]. Collagen forms macromolecules through cross-linking between molecules and enhances the strength and toughness of tissues [28]. In addition, non-collagenous domains present in collagen can self-assemble or assemble with other ECM proteins, providing the complexity of the supramolecular structure. Thus, collagen can form fibers, beaded fibers, anchored fibers, and even collagen networks. Collagen is a protein with a high glycosylation level and a long half-life, and its degradation is crucial in the formation and development of tumor tissues. Matrix metalloproteinases (MMPs) are involved in the physiological and pathological degradation of collagen. In the process of collagen degradation by MMPs, several signal molecules are released from collagen, subsequent altering the mechanical properties and signal transduction in TME [29]. Collagen is a major component of tumor ECM, which affects tumor cell proliferation and intercellular signaling.

Fibronectin, although low expression in tumors, shows multiple functions in ECM [30, 31]. Fibronectin works in cell adhesion, migration, proliferation, blood coagulation, and vascularization [32]. In ECM, fibronectin connects the structural proteins to construct a complete matrix [33]. In addition to binding multiple structural proteins to enhance ECM, fibronectin can also interact directly with other proteins to perform regulatory functions [34]. First, fibronectin is rich in arginine-glycine-asparagine (RGD) sequences to recognize and bind integrins on cell membranes. Therefore, fibronectin works in intracellular signal transduction through induction of integrin attachment. In addition, fibronectin can also interact directly with many growth factors, such as insulin-like growth factor (IGF), fibroblast growth factor (FGF), TGF-β, hepatocyte growth factor (HGF), and platelet-derived growth factor (PDGF) [35]. Therefore, despite the low levels of fibronectin, it still plays a key role in tumor malignant transformation.

Elastin is an important component of elastic fibers, which mainly exists in ligament and vascular wall. Together with collagen, elastin maintains the strength and strength of tissue by resisting it from deforming or breaking. Elastin is highly elastic compared to collagen due to its amino composition and dynamic three-dimensional structure. Laminin proteins, combined with collagen, form the components of the basement membrane and involves in the vascularization during vascular maturation [36]. Laminin is upregulated during epithelialization, providing an interface for epithelial cell adhesion, allowing it to adhere and stretch, therefore alerting tumor metastasis.

Hyaluronic acid (HA) is another major component of ECM, which has important functions, such as regulating vascular wall permeability, promoting wound healing, and material diffusion and transportation [37, 38]. The number of disaccharide repeat units in HA molecule reaches more than 10,000 Da. The long polymer chain forms random entanglement in the solution, and a large number of hydroxyl groups capture water molecules by forming hydrogen bonds, thus increasing the elastic viscosity of ECM [39]. In addition, HA can serve as an important "reservoir" for water, buffered ion exchange, and water and osmotic balance in ECM. Some substances and biomacromolecules have selective permeability due to their charged surfaces and selective domains. Furthermore, HA can be recognized by tumor cells through intracellular signal transduction molecules, such as membrane receptor CD44. This specific recognition plays a crucial role in the migration and invasion of tumor cells [40, 41].

In addition, ECM is an important "catalyst" for the realization of a variety of growth factors. First, ECM can act as a repository for growth factors and signaling molecules for malignant transformation of tumor cells. PDGF can effectively accumulate in ECM after combining with collagen [42]. Heparin binding growth factor 1 (HBGF-1), a growth factor associated with angiogenesis, also binds type I and type IV collagen [43]. Paralkar et al. illustrated that TGF-β could bind with type IV collagen in the basement membrane [44]. Furthermore, ECM can promote the interaction between growth factors and their receptors. Heparin sulfate proteoglycan could promote the interaction between Wnts and Frizzled, thereby stimulating the proliferation of HCC cells [45]. In addition, degradation of ECM contributes to the release of growth factors and cytokines. MMP is overexpressed in tumors, and a large amount of vascular endothelial growth factor (VEGF) will be released after ECM cutting, which promotes tumor development [46].

## Vascular microenvironment

TME is closely related to the generation of tumor angiogenesis; the diameter of the tumor is usually no more than 2 mm in the absence of tumor angiogenesis [47]. When the diameter of solid tumor exceeds 2 mm, it is difficult to obtain enough oxygen and nutrients from the surrounding environment, and tumor cells will secrete angiogenic factors into TME to induce the formation of new blood vessels [48]. Tumor cells directly or indirectly participate in the formation of tumor blood vessels. In hypoxic environment, hypoxia-inducible factor (HIF) directly upregulates the gene expression of VEGF and other angiogenic factors [49]. Marjon et al. found that VEGF mRNA expression level was increased by 10–50 times when the oxygen concentration of tumor cells was reduced from 21 to 0.3% [50]. The mRNA expression of VEGF was relatively high in the necrotic and vascularless areas of tumor tissue. Tumor cells polarize the recruited cells to form tumor-associated stromal cells, promote the expression of vascular growth factor, and indirectly induce the formation of tumor angiogenesis. VEGF can bind to vascular endothelial growth factor receptor (VEGFR) in tumor tissues to activate downstream signaling pathways and change vascular permeability, thereby promoting tumor angiogenesis [51]. Tumor angiogenesis is closely related to tumor growth and metastasis. Inhibition/destruction of tumor angiogenesis microenvironment will be one of the potential tumor treatment strategies.

## Stromal cells

Stromal cells mainly include cancer-associated fibroblasts (CAFs), endothelial cells, and adipocytes. They can perform signal transmission and secrete cytokines to regulate tumor cell proliferation, metastasis, and escape from immune system attack through direct or indirect interaction with tumor cells. It is greatly significant for the diagnosis and treatment of tumors by exploiting the role of stromal cells in tumor genesis, development, and metastasis.

CAFs are derived from fibroblasts and mesenchymal stem cells inherent to local tissues of bone marrow and fat. CAFs exist in all stages of solid malignant tumors. CAFs can differentiate into static fibroblasts and bone marrow-derived mesenchymal stem cells, as well as epithelial cells, smooth muscle cells, pericytes, and adipocytes [52]. Tumor cells can recruit CAFs precursor cells and induce their activation into CAFs. In addition, CAFs can secrete a variety of cytokines and ECM to promote tumor proliferation and metastasis. CAFs also involve in vascular generation, ECM remodeling, immunosuppression, and other exogenous pathways conducive to tumor genesis and development [53]. Generally, CAFs inhibit tumors initially, but as tumors further deteriorate and develop, they transform into tumorigenic cells [54]. Cytokines and chemokines secreted by CAFs have immunosuppressive and activation effects on a variety of lymphocytes, including CD8^+^ T cells, DCs, macrophages, and Tregs. However, CAFs have an immunosuppressive effect against the immune system. The secretion of IL-6, CXCL-9, and TGF-β by CAFs plays a significant inhibitory role in anti-tumor T cell response [55]. CAFs recruit and retain T lymphocytes in tumor tissues through different mechanisms, such as chemokines, cell adhesion molecules, inhibition of immune checkpoint activation, and CD8^+^ T cell exhaustion [56]. In addition, CAFs are the sources of a variety of growth factors, including TGF-β, VEGF, chemokines, and interleukins [57]. Secretion of these factors can promote the transformation of tumor cells, enhance the dryness of existing tumor stem cells, and promote epithelial mesenchymal transformation (EMT) of tumor cells [58].

Macrophages are another kind of stromal cells in tumor tissue with remarkable heterogeneity and variability. Macrophages can transform their functions and phenotypes according to the surrounding environment [59]. Generally, macrophages can be divided into M1 and M2 subtypes [60]. The M1 subtype consists of classically activated pro-inflammatory macrophages that have bactericidal, tumor-suppressive, and antiangiogenic functions. They can be activated through their pattern recognition receptors when recognizing molecular patterns associated with damaged tissues or pathogens, and then produce inflammatory cytokines, including macrophage colony-stimulating factor 1 (M-CSF1) and granulocyte–macrophage–CSF [61]. M2 subtype macrophages can produce anti-inflammatory, immunosuppressive chemokines, and cytokines, but have no cytotoxic activity to tumor cells [62]. Tumor-associated macrophages (TAMs) are induced to differentiate by growth factors and cytokines in tumor tissues [63]. Recruitment and differentiation of TAM macrophages at tumor sites are mainly induced by granulocyte–macrophage colony-stimulating factor (GM-CSF), CCL2, VEGF, IL-6, and IL-8, which are related to hypoxia, acidity, and inflammation of tumor tissues [64]. TME invasion is determined by CC chemokines produced by local lymphoendothelial cells and stromal cells, which have been confirmed in multiple tumor types [65]. TAM, guided by the increasing gradient of chemotactic molecules, infiltrates the hypoxic/necrotic regions of tumors in large quantities and survives by shifting its metabolism to glycolysis [66]. Besides, TAM can produce high levels of TGF-β, blocking the proliferation and killing effects of cytotoxic T cells, while activating immunosuppressive Treg cells [67].

Another important cell type in tumor tissue is adipocytes. Adipocytes in TME can secrete a variety of tumor-related adipocytokines, which works in the occurrence and development of tumors [68]. Due to the influence of hypoxia and high pressure in tumor microenvironment, pathological metabolic disorder occurs in adipocytes, which changes the secretion of adipokines and lipid metabolites around tumor cells, activates signal transducer and activator of transcription 3 (STAT3) and other tumor-related signaling pathways, and accelerates the further development and deterioration of tumor [69].

### Immune cells

Tumor-infiltrating lymphocytes (TILs) are the collective term of all lymphocytes in tumor tissue [70]. TILs are produced by lymphoid organs and circulated to tumor tissues. They are the main cellular components of the host in response to anti-tumor immune response. TILs can be divided into T lymphocytes, B lymphocytes, and natural killer (NK) cells according to its origin and surface markers.

T lymphocytes play a central role in anti-tumor immune response and are the dominant element in TME. Tumor-specific antigen (TSA) can activate highly specific CD8^+^ T cells CD4^+^ T cells and tumor-specific antibodies [71]. CD8^+^ T cells inhibit tumor proliferation by direct lysis of tumor cells or release of IFN-γ and TNF-α [72]. Prolonged exposure to antigens of tumor-specific T cells can lead to their exhaustion. Programmed cell death-1 (PD-1) and T cell immunoglobulin-3 (TIM-3) are considered as immune markers of T cell exhaustion [73]. CD8^+^ T cells may play a functional state of immune activation or immunosuppression based on the balance between costimulatory and coinhibitory signals in the microenvironment [74]. CD4^+^ T cells are mainly including Th1, Th2, Th17, and Tregs. Th1 stimulates the tumor-killing effect of CD8^+^ T cells through cytokines, such as IFN-γ, TGF-β, and IL-2 [75]. Furthermore, it can also promote the activation of macrophages and the maturation of DC cells, while Th2 can assist eosinophils to achieve killing effect [76]. Th17 cells can be converted to Th1 properties and play an anti-tumor role in specific cytokine environments. On the contrary, they are capable to be converted to achieve the properties of Tregs and promote tumor progression in some special conditions [77]. Tregs are a subtype of CD4^+^ T cells to prevent autoimmune diseases. They are recruited to TME by chemokines secreted by tumor cells and macrophages, further inhibiting the anti-tumor immune response [78]. Tregs are activated after recognizing the tumor-associated antigen (TAA) released by damaged tumor cells, effectively inhibiting the activation of TAA-specific effector T cells, releasing a variety of cytokines, and ultimately inhibiting the anti-tumor immune-killing effect [79]. In addition, Tregs can inhibit the action of a variety of other immune cells [80]. The high infiltration rate of Tregs in tumors is closely associated with local recurrence, rapid tumor progression, and high sentinel lymph node metastasis rate [81]. The ratio between different T cell subsets is also an important indicator of tumor occurrence and development. CD8^+^/FoxP3^+^ and CD8^+^/CD4^+^ ratios are the most commonly used indicators, indicating the strength of anti-tumor immune activity. In order to escape immune attack, tumor cells inhibit the role of tumor-specific T cells and induce the activation of immunosuppressed Tregs, thus reducing the ratio of CD8^+^ T cells/Tregs [82].

NK cells, derived from bone marrow lymphoid stem cells, differentiate and mature in bone marrow and thymus. They can kill tumor cells nonspecifically without dependence on major histocompatibility complex (MHC) [83, 84]. NK cells participate in the regulation of adaptive immune response through interaction with DC cells and arise a strong cytotoxic immune response against tumor cells [85]. Tumor cells resist NK cell killing by releasing TGF-β, downregulating antigenic expression and increasing MHC I [86]. In addition, Tregs can also compete with NK cells for IL-2, thereby inhibiting NK cell activity. Tumor-infiltrating NK cells can limit blood metastasis of tumor cells [87].

B lymphocytes are derived from bone marrow pluripotent stem cells, accounting for 15–20% of all infiltrating lymphocytes [88]. B lymphocytes can be differentiated into plasma cells under antigen stimulation, which can secrete antibodies and activate humoral immune response of the body. The infiltration of B lymphocytes is associated with primary melanoma proliferation, reduced risk of metastasis, and prolonged survival [89]. The mechanism of the role of tumor-infiltrating B lymphocytes in anti-tumor immune response is still unclear and needs to be further studied.

Dendritic cells (DCs) are specialized antigen presenting cells that can absorb, process, and present antigens and initiate specific immune responses mediated by T lymphocytes. DCs can recognize tumor antigens to regulate the innate and adaptive immunity [90]. DCs cross-presents antigens to CD8^+^ T lymphocytes through MHC I molecules after obtaining tumor-associated antigen, thus inducing the hosts’ specific anti-tumor response. DCs can also directly participate in cytotoxic immune responses by activating NK cells [85]. In TME, IL-10, TGF-β1, VEGF, and other factors secreted by tumor cells or TAMs can inhibit the maturation of DCs, thus avoiding the host's anti-tumor immune response [91]. Immature/tolerant DCs can regulate tumor angiogenesis and contribute to tumor proliferation [92]. Mature DCs are mainly distributed around the tumor and are related to the activation state of T lymphocytes, tumor size, and patient survival. The number and distribution of mature DCs can be used as an indicator of the efficacy of immunotherapy for tumor patients [93].

### Immune checkpoints

Immune checkpoint (IC) refers to programmed death receptors and their ligands. Immune checkpoint blockade (ICB) therapy based on programmed death receptor (PD-1) and its ligand (PD-L1) can improve the host immune system's aggression against tumor cells by inhibiting the binding of PD-1 and PD-L1 [94]. The basic principle of ICB therapy is based on the activation mechanism of immune cells called T cells. Programmed death receptors are expressed on the surface of T cells and their ligands are expressed on tumor cells and myeloid suppressor cells. The combination of programmed death receptor and its ligand can cause T cells to fail to kill tumor cells, so that tumor cells can escape the immune surveillance of the host. Therefore, ICB therapy based on PD-1/PD-L1 can improve the host immune system's aggression against tumor cells by inhibiting the combination of programmed death receptor and its ligand [95]. Cytotoxic T lymphocyte antigen-4 (CTLA-4) is another classical immune checkpoint and can be phosphorylated to activate phosphoinositide 3-kinase (PI3K) pathways to achieve dephosphorylation of the CD3ζ chain, which limits the signaling potential of the TCR. There are also other important ICs, such as lymphocyte activation gene 3 (LAG-3), TIM-3, Exostosin-like glycosyltransferase 3 (EXTL3), V-domain Ig suppressor of T cell activation (VISTA), and indoleamine 2,3-dioxygenase (IDO) [96–98]. In 2011, the US Food and Drug Administration (FDA) approved the first ICB antibody Ipilimumab for advanced melanoma, and tumor immunotherapy has received unprecedented attention and development since then [99]. In 2018, the Nobel Prize in Physiology or Medicine was awarded to James Allison and Tasuku Honjo for their outstanding contributions to ICB therapy of cytotoxic T lymphocyte antigen-4 (CTLA-4) and PD-1/PD-L1 signaling pathways, respectively [100]. Together, ICB therapies open the possibility of including TME-based markers for selecting patients who are likely to respond to these specific therapies and pave the way to personalized medicine in oncology [101].

## Exosomes

In TME, cell-to-cell communication is one of the important factors affecting tumor progression. Exosomes, as important signal carriers of cell-to-cell communication in TME, have attracted much attention in recent years [102]. Exosome refers to the extracellular sac with lipid bilayer membrane structure formed by cells under physiological and pathological conditions through “endocytosis-fusion-efflux” and other regulatory processes [102], mainly containing proteins, mRNA, micro-RNA, and cytokines. Exosomes are involved in many important pathophysiological processes, including remodeling TME, mediating specific cellular communication, regulating angiogenesis, immune escape, and distal metastasis in TME, thereby regulating tumor genesis and development [103]. There are conflicting mechanisms between immune promotion and immunosuppression of exosomes. Tumor-derived exosomes can both stimulate and inhibit specific and non-specific immune responses [104]. Exosomes containing tumor-associated antigens can be effectively absorbed by antigen-presenting cells, such as DCs, resulting in anti-tumor effects [105]. In addition, exosomes produced by premetastatic tumors trigger a broad innate immune response through immune monitoring, leading to the elimination of cancer cells in the premetastatic niche [106]. Exosomes are involved in immune suppression. Exosomes isolated from plasma of head and neck cancer patients can effectively induce apoptosis of CD8^+^ T cells, inhibit proliferation of CD4^+^ T cells, and enhance immunosuppression of regulatory T cells. Razzo et al. [107] confirmed that a single intravenous injection of tumor-derived exosomes was sufficient to accelerate tumor progression in mice with OSCC precancerous lesions and reduced the migration of immune cells to tumors.

## Physicochemical microenvironment

Due to the rapid proliferation of tumor cells, glucose and energy are consumed seriously, and oxygen is exhausted, which leads to the increase of lactic acid level, interstitial pressure, and acidification of microenvironment in tumor tissues [108].

In normal tissues, extracellular pH is tightly regulated, while dysregulation of pH often occurs in tumor microenvironment (pH 6.7 ~ 7.1). It is mainly due to anaerobic glycolysis and lactic acid produced by tumor cells. The pKa value of lactic acid is approximately 3.9, which is always lower than the pH value of tumor microenvironment, so it usually exists in the form of lactate and H^+^ ions. In addition, CO_2_, produced by respiration synthesizes, can be metabolized into H_2_CO_3_ under the action of carbonic anhydrase, which can be dissociated into HCO_3_^−^ and H^+^ ions [109]. Moreover, abnormal blood perfusion and the absence of functional lymphatic vessels further limit acid excretion from the TME. TME can promote degradation of ECM, leading to local invasion of tumor cells [110]. Acidity is a common characteristic in tumor microenvironment, while delivery systems targeting low pH of tumor tissues can be developed to selectively deliver drugs to tumor cells, so as to achieve specific killing of tumors [111].

Hypoxia is another important feature of TME, which can accelerate the survival and invasion of tumor cells [112]. Hypoxic is often considered as a major adverse driver of immunotherapy resistance, resulting in gene mutations, transformation of signal transduction, and activation of effector molecules [113, 114]. In a hypoxic environment, HIF-1α loses its degradation properties and binds to HIF-1β to form transcriptionally active dimers [115]. These dimers can interact with transcriptional coactivators to induce transcription regulation of target genes associated with immune escape. Hypoxia regulates tumor immunomodulatory process: (1) Hypoxia can recruit various immunosuppressive cells, mainly affecting Tregs, TAM, and marrow-derived suppressor cells (MDSCs), therefore promoting the immune tolerance of tumor cells; (2) Hypoxia can upregulate the expression of inhibitory immune checkpoint on the surface of tumors, thus inhibiting the specific function of T cells and evading host immune surveillance [116]; (3) Autophagy can be induced in a hypoxic environment. Autophagy is a key regulator of cell viability, clearing dysfunctional organelles and preventing the progression of cancer [117]. Nevertheless, advanced tumors can degrade aggregates of harmful proteins [118], remove ROS [119], and promote recruitment of Treg cells [120] by autophagy, ultimately achieving immune escape; (4) Hypoxic-mediated immune effector cell inhibition. Immune effector cells, such as T cells, DCs, and NK cells, were inhibited in hypoxic environment. Hypoxia not only inhibits the ability of CTL to melt tumor cells but also greatly affects the proliferation and activity of CTL and ultimately fails to provide enough CTLs to eliminate tumor cells [121]. Downregulated expression of costimulatory molecules of DCs in hypoxic environment failed to effectively induce T cell activation [122].

In summary, the occurrence, development, metastasis, and recurrence of oral cancers are inseparable from specific tumor immune microenvironment. With the deep understanding of TME, the specific cancer immunotherapy will dramatically alter the landscape in fundamental researches and clinical application of oral cancers.

## Immunotherapy for oral cancer

The clinical treatment of tumor is mainly surgery, radiotherapy, and chemotherapy [123]. Surgical resection is currently the preferred treatment for many early and intermediate stage cancers, and can be treated by partial or total removal of the lesion site and surrounding tissue. However, surgical resection is traumatic and limited. It is generally applicable to the primary lesion, unable to eliminate the metastatic cancer cells and unable to touch the cancer cells in the blood circulation. In addition, surgical treatment of patients with higher physical requirements; otherwise it will accelerate the spread and metastasis of cancer cells due to reduced immunity [124]. Radiation therapy is to use radiation to irradiate cancerous tissue and destroy the DNA chain of cancer cells to achieve local treatment of cancer. Radiotherapy usually has a long treatment cycle, high cost, and will produce a variety of complications, damage the body function, and weaken the body immunity [125]. Chemical treatment usually adopts systemic drug treatment, the lack of selectivity, will affect normal cells, side effects, although the molecular targeted drugs and new preparations of nanometer drug has made significant progress, but the treatment effect is not satisfactory, and the cancer is easy to produce resistance and resistance to chemotherapy drugs, tends to increase treatment difficulty [126]. On the other hand, the high osmotic pressure of tumor tissue makes it difficult for chemotherapy drugs to penetrate into the tumor site, which further limits the efficacy of chemotherapy. Generally, traditional Chinese Medicine plays an auxiliary role in reducing complications from chemotherapy or other treatments.

Although the above-mentioned traditional tumor treatment methods can temporarily curb the development of tumor, they cannot fundamentally solve the problems of tumor recurrence and metastasis. Moreover, oral cancer shows a high mutation rate associated with DNA damage caused by smoking and alcohol [127]. Therefore, it is urgent to develop new cancer treatment strategies to achieve efficient tumor treatment while reducing toxicity and improving the quality of life of patients.

**Fig. 2:**
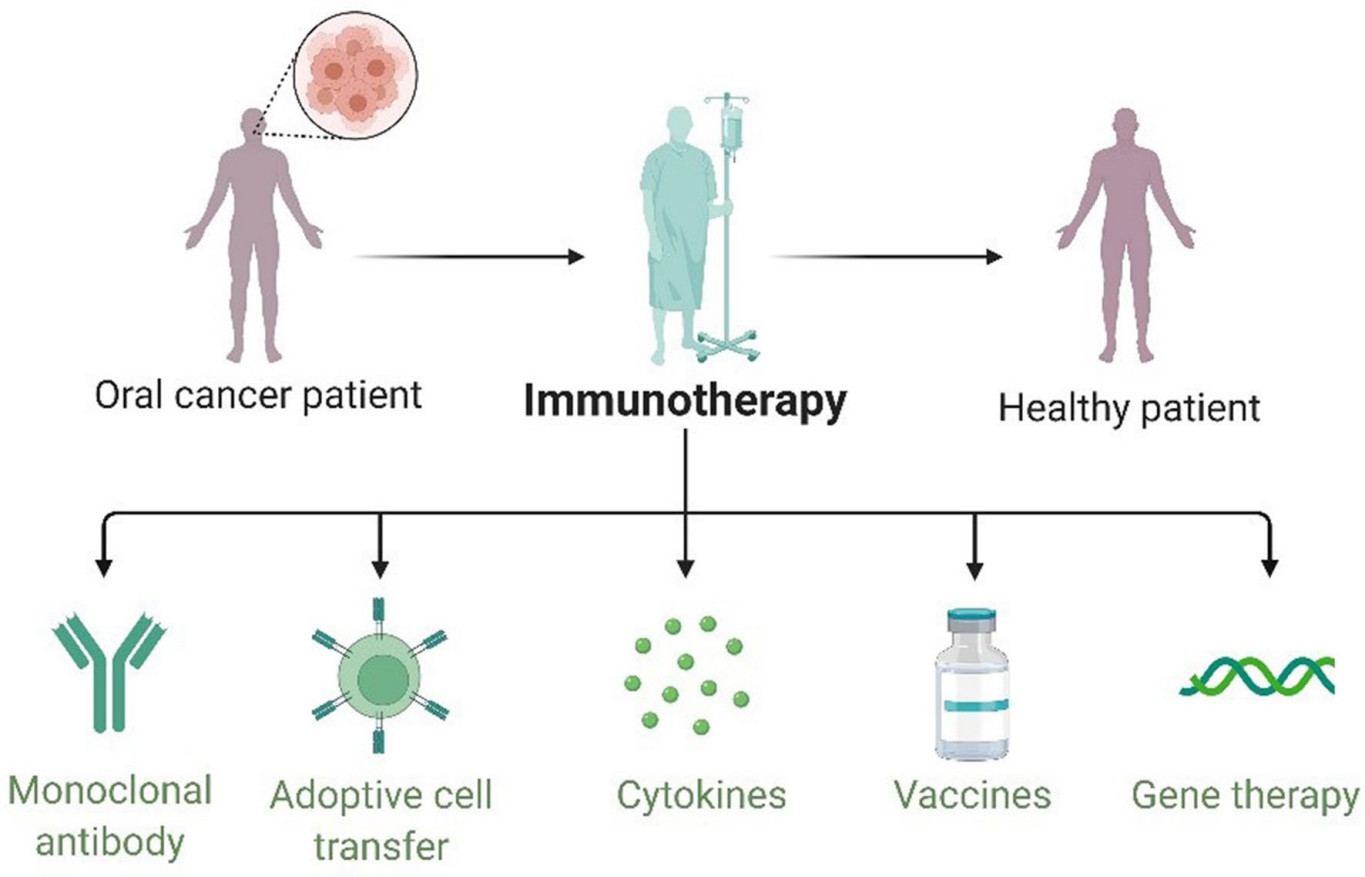
Immunotherapy strategies for oral caners

Immunotherapy is a new strategy for tumor therapy, which applies biotechnology and immunological methods to improve the specific immune response to tumor [128, 129]. Tumor immunotherapy was awarded as the most important scientific breakthrough by Science in 2013 due to its excellent efficacy and innovation [17]. In 2020, the Nobel Prize in Chemistry was awarded to French microbiologist Emmanuelle Charpentier and American biologist Jennifer Doudna for their "development of genome editing methods." Immunotherapy has outstanding application value in the field of tumor therapy, including adoptive cell immunotherapy, antibody-based therapy, cytokine therapy, tumor vaccines therapy, and gene therapy (Fig. 2).

## Monoclonal antibody-based therapies

Monoclonal antibodies (mAbs) are highly homogeneous antibodies produced by a single B cell clone that bind to a specific epitope. In 1975, the mAbs technology were first developed by Köhler and Milstein by using B cell hybridomas, with unlimited reproductive ability as well as secreting specific antibodies [130]. In 1997, Rituximab was the first mAb approved by FDA for the immunotherapy of B cell non-Hodgkin’s lymphomas by targeting CD20 [131]. Benefiting from the rapid development of immunology and protein engineering, mAbs immunotherapy is currently the fastest growing immunotherapy. These drugs can recruit T cells to the tumor site, directly target the tumor cells, change the host's response to the tumor, and thus inhibit or even eliminate the tumor [132, 133]. Furthermore, mAbs are capable to achieve anti-tumor angiogenesis by inhibiting the oxygen supply and nutrient transport of tumor cells [134]. After decades of research and development, mAbs have shown unique advantages and made great progress in the field of cancer and other major diseases, and they are also the fastest growing and most promising development direction in the field of medicine. There are several ongoing studies with mAbs in oral cancers related to the key words (“oropharyngeal cancer” OR “oral cancer” OR “oral cavity cancer”) and (“Monoclonal antibodies” OR “Monoclonal antibody” OR mAbs OR mAb) from ClinicalTrials. Gov. As shown in Table 1, there are 9 clinical trials, which include 4 anti-angiogenesis mAbs and 5 immune checkpoint inhibitors. In addition, there are several other targets were also important for monoclonal antibody-based oral cancer clinical treatment, including receptor tyrosine-protein kinase erbB-3 [135], nucleophosmin [136], and c-Met protein with tyrosine kinase activity [137]. Moreover, monoclonal antibodies have shown great potential in the prevention of oral cancer. Oral proliferative verrucous leukoplakia (OPVL) is a rare refractory leukoplakia, which is a precancerous lesion of oral cancer. It is likely to develop into oral squamous cell carcinoma or verrucous carcinoma. Nivolumab shows potential to shrink the white lesions in the participant's mouth and reduce cancer risk and is undergoing clinical trial for prevention of OPVL (NCT03692325).

**Table 1:** Monoclonal antibody-based trials for oral cancers

Product name	Sponsor	Target	Estimated date of completion	Phase	Status	Identifier
Cemiplimab	ISA Pharmaceuticals	PD-1	November 2024	II	Recruiting	NCT04398524
Bevacizumab	National Cancer Institute (NCI)	VEGF-A	March 2010	I	Completed	NCT02002182
Anti-EGFR monoclonal antibody	Fudan University	EGFR	December 2020	II	Recruiting	NCT04508829
Nivolumab	Medical University of South Carolina	PD-1	November 2021	I/II	Completed	NCT03021993
Cetuximab	Eastern Cooperative Oncology Group	EGFR	April 2004	III	Completed	NCT00003809
Sintilimab	Sun Yat-Sen Memorial Hospital of Sun Yat-Sen University	PD-1	October 2027	II	Not yet recruiting	NCT05000892
Toripalimab	Sun Yat-Sen Memorial Hospital of Sun Yat-Sen University	PD-1	June 2027	II	Not yet recruiting	NCT04825938
Anti-OX40 antibody	Providence Health & Services	OX40	October 2022	I	Active, not recruiting	NCT02274155
Bevacizumab	M.D. Anderson Cancer Center	VEGF	March 2022	I	Active, not recruiting	NCT01552434

*PD-1* Programmed cell death-1; *VEGF-A* Vascular endothelial growth factor A; *EGFR* Endothelial growth factor receptor; *OX40 (CD134)* A member of the tumor necrosis factor family of receptors

Tumor microenvironment can induce immune tolerance of tumor-specific T cells, weaken T cell function, and lead to immune escape [138]. Among them, the main strategy of tumor cells to achieve immune escape is overexpression of immune checkpoint molecules. Immune checkpoint inhibitors (ICIs) are one of the most promising immune-based interventions. Blockade of immune checkpoint signaling pathway is capable to activate anti-tumor immune response and shows extraordinary clinical application value. The PD-1/PD-L1 blockade has been identified as a potential therapeutic pathway to inhibit the activation of T cells and cytokine production in tumor cells [139, 140]. So far, four mAbs have been approved against PD-1/PD-L1 blockade in clinical studies for oral cancer treatment, including cemiplimab (NCT04398524), nivolumab (NCT03021993), sintilimab (NCT05000892), and toripalimab (NCT04825938). OX40 is a costimulatory molecule that can enhance T cell immunity. MEDI6469, an anti-human OX40 neoadjuvant antibody, was performed in a phase I clinical trial (NCT02274155) prior to surgery. The results demonstrated that anti-OX40 mAb was safe and could induce the activation and proliferation of T cells in hosts, suggesting a potential clinical strategy [141]. Furthermore, mAbs blocking other immune checkpoint receptors, particularly CTLA-4, TIM-3, LAG-3, and IDO, have attracted worldwide attentions due to their impressive results [142]. Nevertheless, the overall response rate of mAbs in the treatment of oral cancers should be further improved.

Tumor angiogenesis is a complex process including vascular endothelial matrix degradation, endothelial cell migration and proliferation, vascular branching, and formation of new basement membrane [143]. Due to abnormal structure and imperfect vascular matrix of tumor vessels, tumor cells can directly penetrate into blood vessels to form metastasis in distant sites without a complex invasion process [144]. Benign tumors have little angiogenesis and slow blood vessel growth, while malignant tumors demonstrate dense angiogenesis and rapid growth. Therefore, angiogenesis plays an important role in the development and metastasis of tumor, and inhibition of this process can significantly prevent the development and metastasis of tumor tissue [145]. As an anti-VEGF mAb, Bevacizumab was first approved by FDA for metastatic colorectal cancer treatment in 2004 and spread to non-small cell lung cancer, glioblastoma, metastatic renal cell carcinoma, advanced cervical cancer, drug-resistant ovarian cancer, metastatic hepatocellular carcinoma, and oral cavity carcinoma et al. [143]. There are 2 clinical trials of Bevacizumab for oral cancer. National Cancer Institute (NCI) has completed its primary outcome measure on March 2010 in Phase I clinical trial (NCT00023959). M.D. Anderson Cancer Center is ongoing its Phase I clinical trial (NCT01552434). This study will evaluate the side effects and best dose of Bevacizumab alone or in combination with other drugs. Based on EGFR-targeted therapy, two clinical trials were performed in the treatment of oropharyngeal carcinoma. Eastern Cooperative Oncology Group evaluated the effectiveness of cetuximab with cisplatin for metastatic or recurrent head and neck cancer patients by the randomized double-blinded phase III trial (NCT00003809). Wang et al. carried out a prospective phase II trial to evaluate the efficacy and safety of anti-EGFR monoclonal antibody combined with intensity-modulated radiation therapy in locally advanced oropharyngeal carcinoma (NCT04508829).

## Adoptive cell transfer

Adoptive cellular immunotherapy (ACI) is an important method in tumor biotherapy. ACI refers to the transfer of tumor patients with anti-tumor activity of immune cells, directly killing tumor or stimulate the body’s immune response to kill tumor cells, so as to achieve the purpose of tumor treatment [146]. ACI can be used in clinical treatment of cancer patients alone, more importantly, as a supplement to surgery, radiotherapy, and chemotherapy. Currently, the clinical research and application of ACI therapy cells mainly include lymphokine-activated killer (LAK) cells, chimeric antigen receptor T (CAR-T) cells, CD3AcAb-activatived killer (CD3AK) cells, cytokine-induced killer (CIK) cells, and DC cells [147].

CAR-T cell therapy, one of the most important ACI, has obtained obvious success against hematological tumors by targeting tumor antigens [148]. To obtain CAR-T cells, T cells are isolated from patient’s peripheral blood, then the CARs are transduced by genetic engineering technique to achieve the targeted recognition of tumor cells. After cell amplification, the autologous CAR-T cells are infused back into patients [149]. In 1993, Zelig Eshhar et al. first proposed CAR-T treatment [150]. Over the next 3 decades, this technology has gone through five generations and gradually matures. The latest generation of CAR-T cells contains CD3ζ chain for signal transduction, a co-stimulatory molecule for activation and proliferation, and intracellular domains of cytokine receptors [143, 151]. Mei et al. constructed MUC1-targeting CAR-MUC1-IL22 T cells and validated the cytotoxic function in human tongue squamous carcinoma cells. These cells showed an effective cytotoxic function against tumor cells [152]. Chan and co-workers screened out CD70 CAR-T cells among nine potential targets and evaluated their anti-tumor effect in human oral squamous cell carcinomas. The results demonstrated the specific recognition and efficient elimination of CD70-positive cancer cells and provided a potential CAR-T target [153]. Recently, Yang et al. constructed an all-in-one CAR-T cells by coupling anti-human epidermal growth factor receptor 2 (anti-HER2) CAR signaling and clustered regularly interspaced short palindromic repeats interference (CRISPRi)-mediated PD-1 gene suppression. These symphysis CAR-T cells reversed PD-1/PD-L1 immune checkpoint inhibition and promoted the persistence and effectiveness against HER2-expressing human head and neck squamous cell carcinoma [154].

**Fig. 3:**
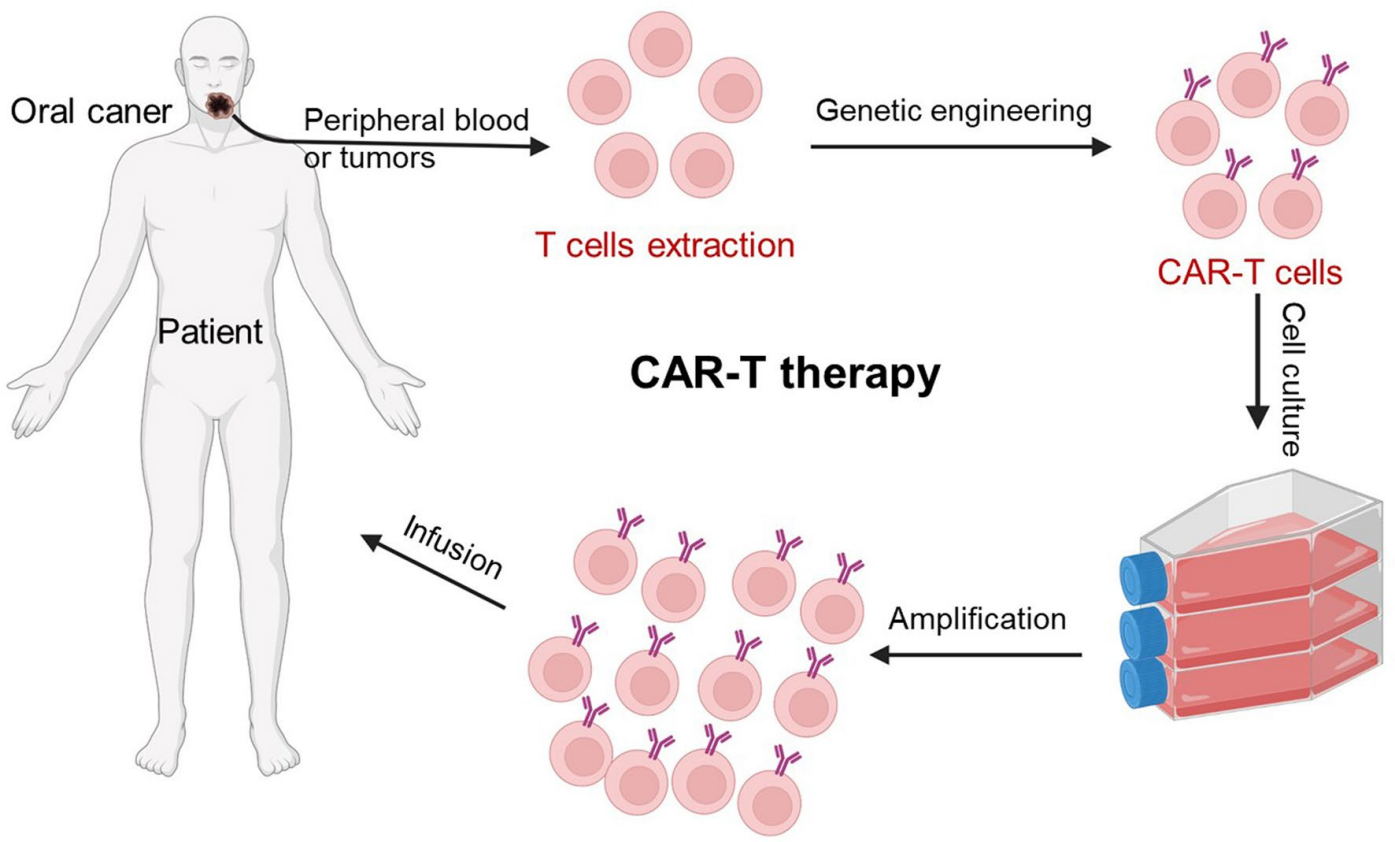
Schematic diagram of CAR-T therapy

Although CAR-T therapy has obtained amazing achievements (Fig. 3), the potential severe toxicities still limit their widespread application, mostly cytokine storms, which can induce high fevers and even multi-organ dysfunction [155]. In addition, the infiltration of solid tumors is another major obstacle for CAR-T therapy. Therefore, degradation of tumor extracellular matrix or regulation of tumor microenvironment are expected to be the effective ways to improve the infiltration of CAR-T cells [156]. Significantly, the immunosuppressive microenvironment of tumors further hinders the effectiveness of CAR-T therapy. Combining immunosuppressive microenvironmental regulation strategies with CAR-T cells may provide a synergic anti-tumor therapeutic effect [157].

## Cytokine therapy

Cytokines are generally produced by stimulated immune cells and shows high efficiency. They can directly stimulate immune effector cells and stromal cells at the tumor site and enhance the killing effect of immune cells [158]. Recently, there are several cytokine drugs approved by FDA, such as high-dose IL-2 for the treatment of melanoma and kidney cancer [159], and IFN-α for the adjuvant treatment of stage III melanoma [160]. IL-12 works on activated T and NK cells and has a wide range of biological activities, acting on lymphocytes by regulating activators of the transcriptional protein STAT4. IL-12 is required for T cell independent induction of IFN-γ and plays an important role in the differentiation of Th1 and Th2 cells [161]. Yang and colleagues illustrated that the receptor of IL-12 family member (IL-23) contributed for the tumor lymph node metastasis of oral cancer [162]. IFN-γ belongs to type II interferon, which is mainly produced by NK and NKT cells and has anti-proliferative effect on transformed cells, and can strengthen the antiviral and anti-tumor effects of type I interferon [163]. Ma et al. detected the decreased expression of IFN-γ mRNA and protein in oral cancer tissues, which was negatively correlative with IFN-γ methylation rate, involving in the process of tumorigenesis of oral cancer [164]. GM-CSF is the earliest found cytokine that has effects on DC cells. GM-CSF induces the differentiation of bone marrow dendritic cells (BMDCs), promotes the immune response of Th1 cells, induces angiogenesis, and influences the development of allergic inflammation and autoimmune diseases [165]. TNF-α belongs to the TNF superfamily of cytokines, which is a multifunctional molecule regulating biological processes. TNF-α can not only kill tumor cells directly but also induce immature DC to differentiate into mature DC [166]. There are several ongoing studies with mAbs in oral cancers related to the key words (“oropharyngeal cancer” OR “oral cancer” OR “oral cavity cancer”) and (“cytokine therapy” OR cytokines OR IFN-α OR IL-2 OR TNF-α) from ClinicalTrials. Gov. As shown in Table 2, there are 8 clinical trials, which include 4 IFN-α, 3 interleukin, and 1 salivary cytokine. With the further study of cytokines, more factors will be applied to tumor immunotherapy.

**Table 2:** Cytokine therapy clinical trials for oral cancers

Cytokines	Sponsor	Country	Estimated date of completion	Phase	Status	Identifier
IL-12	NYU Langone Health	United States	May 2009		Completed	NCT00899821
IL-12 or GM-CSF	National Cancer Institute (NCI)	United States	May 2007	II	Completed	NCT00019331
PEG-IFN-α-2b	M.D. Anderson Cancer Center	United States	March 2006	II	Completed	NCT00276523
IL-2 gene	H. Lee Moffitt Cancer Center and Research Institute	United States	June 2004	II	Completed	NCT00006033
IFN-α	Eastern Cooperative Oncology Group	United States	May 2009	III	Completed	NCT00054561
IFN-α	Hoag Memorial Hospital Presbyterian	United States	February 1999	II	Completed	NCT00002506
Salivary cytokines	University Hospital, Basel, Switzerland	Switzerland	January 202		Completed	NCT02807519
PEG-IFN-α-2b	Dartmouth-Hitchcock Medical Center	United States	November 2002	I	Completed	NCT00014261

*IL-12* interleukin-12; *IFN-α* interferon alfa

## Tumor vaccines

Tumor vaccine is a specific, safe, and tolerable cancer treatment strategy. Currently, FDA has approved two preventive vaccines for the treatment of human papillomavirus (HPV) and hepatitis B virus, which can cause liver cancer [167]. HPV is etiologically involved in cervical cancers mainly through inactivation of tumor suppressor proteins, such as p53 and pRB. HPV is consistently and more frequently detected in cancers of the oropharynx and tonsil than at other head and neck sites [168]. In 2010, PROVENGE (Sipuleucel-T), an immune-based vaccine, was approved by FDA as the first therapeutic oncology vaccine for prostate cancer [169]. In addition, multiple tumor vaccines combined with checkpoint blocking modulators or cytokine therapies are evaluated in clinical trials with potential clinical application in solid tumors or metastatic tumors [170].

The anti-tumor process of tumor vaccines as follows: (1) Tumor-specific antigens are absorbed by antigen-presenting cells and further processed by DCs into short peptides that facilitate the presentation of MHC molecules, which are presented to initial CD8^+^ and CD4^+^ T cells by MHC I or MHC II, respectively. Antigenic peptides are recognized by TCR on the surface of T cells and provide the first signal of activation to T cells. (2) DCs are further activated and the expression of costimulatory molecules (B7 molecules) on cell surface is upregulated. The B7 molecules can bind to the CD28 molecule on T cells, providing a second signal for T cells to activate. (3) After the initial activation of CD8^+^ and CD4^+^ T cells, CD8^+^ T cells further proliferate and differentiate into effector T cells with tumor-killing ability, while CD4^+^ T cells mainly differentiate into Th1 and Th2 cells under the stimulation of different cytokines to assist cellular and humoral immunity of the host, respectively. (4) Effector T cells circulate and infiltrate into tumor tissues, recognize, and kill tumor cells. The killed tumor cells can further provide tumor antigens, amplifying the immune response [171, 172].

There are mainly three types of tumor vaccines, including inactivated vaccines, protein/polypeptide vaccines, and nucleic acid-based vaccines [173]. Live tumor cells can produce immunosuppressive cytokines and may form new tumors in vivo [174]. However, inactivated vaccines use TAAs from tumor cells excised from patients (autologous tumor cells) or cultured tumor cells (allogeneic tumor cells) as antigen sources, which are physically or chemically inactivated and added with adjuvants as tumor vaccines [175, 176]. A major advantage of using tumor cells as an antigen source is that there are a series of mutated tumor antigens on tumor cells that are more immunogenic than generic tumor antigens and can synergistically enhance the body to produce specific immune response [174]. Protein/polypeptide vaccines are mainly derived from protein fragments or intact proteins specifically expressed by tumor cells. Polypeptide vaccines are generally obtained by chemical synthesis, saving time, and cost [177]. Protein vaccines are achieved by recombinant protein technology [178]. Many preclinical and clinical trials have confirmed that protein/polypeptide vaccines are safe and have high clinical application value [177, 179]. However, protein/polypeptide vaccines usually target only one or more TAAs, so a combination of antigens is required to induce stronger tumor-specific T cell responses [37, 180, 181]. More than 70 percent of oropharyngeal cancers are associated with human papillomavirus infection. Therefore, HPV vaccine can prevent and treat oropharyngeal cancer [182]. In 2020, FDA approved an expanded indication of Gardasil 9 for the prevention of oropharyngeal and other cancer caused by HPV [183]. These vaccines were mainly developed against E6 and E7 proteins in clinical trials. Vaccines based on nucleic acids (DNA or RNA) are a promising vaccine platform. In the 1990s, scientists discovered that plasmid DNA can induce a strong antibody response against the antigens it encodes [184]. Nucleic acid vaccines have many advantages. First, nucleic acid vaccines allow simultaneous delivery of multiple antigens covering various TAAs or tumor mutations, while simultaneously activating the body's humoral and cellular immune responses, increasing the possibility of overcoming vaccine resistance. Secondly, unlike many peptide-based vaccines, DNA vaccine, encoding full-length tumor antigen, allows the APC cells present at the same time or cross-presented with class I and class II specific human leukocyte antigen (HLA) in patients with multiple epitopes, therefore stimulating a wider range of T cell response [185]. More importantly, nucleic acid vaccines are non-infectious and do not contain contamination from protein or viral sources during production and are therefore considered to be well tolerated for both prevention and treatment [173, 186]. Messenger RNA (mRNA) vaccines are an attractive alternative to DNA vaccines that have emerged in recent years for infectious disease prevention and anticancer therapy. On 18 November 2020, Pfizer announced the results of a Phase III clinical trial of BNT162b2 mRNA vaccine jointly developed with BioNTech in Germany, which showed a 95% protective efficiency. On December 31 2020, the World Health Organization (WHO) designated Pfizer/BioNTech's mRNA vaccine as available for emergency use, the first COVID-19 vaccine authorized by WHO. In addition to helping the world fight an urgent pandemic, the mRNA vaccine has greatly advanced the field of vaccine development. In a sense, the novel Coronavirus pandemic has hastened the development of mRNA vaccine, ushering in a new era of vaccine development. It is a predictable event that such vaccines, which can be produced quickly and cheaply, will soon appear in the field of cancer treatment, bringing benefits to human health [186, 187]. However, the problems of mRNA instability, low in vivo delivery efficiency and high innate immunogenicity still need to be further solved [188].

There are several ongoing studies with cancer vaccination in oral cancers related to the key words (“oropharyngeal cancer” OR “oral cancer” OR “oral cavity cancer”) and vaccine from ClinicalTrials. Gov. As shown in Table **3**, there are 17 clinical trials, which include 10 HPV antigen-related vaccine trials, 4 protein/polypeptide-based vaccine trials, 2 vaccine-combined monoclonal antibody therapy, and 1 DC-based vaccine. In general, HPV antigen-related vaccines and combined therapies with other strategies are the primary strategies for oral cancers. In addition, the development of neoantigen and nucleic acid vaccine will be the potential prospects for oral cancer clinical treatment.

**Table 3:** Tumor vaccination clinical trials for oral cancers

Vaccines	Sponsor	Nationality	Estimated date of completion	Phase	Status	Identifier
HPV Vaccination	Oslo University Hospital	Norway	February 2017		Completed	NCT02934724
ADXS-HPV	Andrew Sikora	United States	August 2023	II	Active	NCT02002182
Utomilumab and ISA101b	M.D. Anderson Cancer Center	United States	June 2022	II	Active	NCT03258008
HPV Vaccine PRGN-2009 Alone or in Combination with Anti-PD-L1/TGF-Beta Trap (M7824)	National Cancer Institute (NCI)	United States	October 2023	I/II	Recruiting	NCT04432597
HPV Vaccination	Regenstrief Institute, Inc	United States	June 2015		Completed	NCT02551887
HPV Vaccination	Regenstrief Institute, Inc	United States	May 2016		Completed	NCT02558803
HPV Vaccination	Boston Medical Center	United States	April 2019		Completed	NCT03346915
HPV Vaccination	National Cancer Institute (NCI)	United States	July 2022		Active	NCT00867464
Recombinant Fowl Pox Vaccine rF-CEA (6D)/TRICOM With GM-CSF	National Cancer Institute (NCI)	United States	January 2013	I	Completed	NCT00028496
TheraT® Vectors combined with chemotherapy	University of Chicago	United States	January 2026	I/II	Not yet recruiting	NCT05108870
PDS0101 + NHS-IL12 + M7824	National Cancer Institute (NCI)	United States	January 2023	I/II	Recruiting	NCT04287868
Tumor-specific mutated Ras peptides and IL-2 or GM-CSF	National Cancer Institute (NCI)	United States	May 2007	II	Completed	NCT00019331
HPV Vaccination	National Cancer Institute (NCI)	United States	April 2015	I	Completed	NCT00019110
HPV Vaccination	University of Birmingham	United Kingdom	September 2015		Completed	NCT01330147
Recombinant fowlpox-TRICOM	National Institute on Deafness and Other Communication Disorders (NIDCD)	United States	April 2015	I	Completed	NCT00021424
HPV16-E711-19 Nanomer	Dana-Farber Cancer Institute	United States	May 2023	I/II	Active	NCT02865135
DCs loaded with wild-type p53 peptides with T-helper peptide epitope	Robert Ferris	United States	March 2014	I	Completed	NCT00404339

*CEA* Carcino-embryonic antigen; *GM-CSF* granulocyte/macrophage colony-stimulating factor; *HPV* human papillomavirus; *IL-12* interleukin-12; *PD-L1* programmed cell death-ligand 1; *Ras* Rat sarcoma; *TGF-β* transforming growth factor-β; *TRICOM* Triad of costimulatory molecules

## Gene therapy

Gene therapy can be regulated at the nucleic acid level to achieve the purpose of tumor treatment, including therapeutic genes, nucleic acid vaccine, RNA interference, and gene probe technology [189–192]. Gene therapy can also target the genes those are involved in the immune system interactions. Oncolytic virus is a natural or genetically engineered virus. It belongs to a special gene therapy method that can selectively replicate in tumor cells to cause tumor cell lysis and activate the hosts’ immune system. Oncolytic virus mediates anti-tumor activity mainly through the following ways: selectively replicating in tumor cells, leading to tumor lysis. The tumor-associated antigen released by lysis activates the immune response of the host, thereby removing tumor cells. Viral infection also causes tumor cells to release cytokines, which in turn clear metastatic tumors [193]. In addition, gene therapy strategies targeting immune-related genes of oral cancer are also an important direction for future research. Currently, there are several ongoing studies based on gene-based immunotherapy in oral cancers. As shown in Table 4, there are 5 clinical trials, involving cytokines, Car-T, and gene vaccines. With the continuous innovation of molecular biology, cell biology, and clinical medical technology, gene therapy will play an important role in the process of overcoming the intractable tumor and is expected to become a routine strategy for tumor treatment in the future [194].

**Table 4:** Gene therapy-based clinical trials for oral cancers

Product name	Sponsor	Nationality	Estimated date of completion	Phase	Status	Identifier
IL-12 gene medicine	Dana-Farber Cancer Institute	United States	November 2000	I/II	Completed	NCT00004070
Allovectin-7®	Vical	United States	June 2002	III	Completed	NCT00050388
E6 TCR gene therapy	National Cancer Institute (NCI)	United States	June 2016	I/II	Completed	NCT02280811
TheraT® expressing HPV-specific antigens	Hookipa Biotech GmbH	United States	June 2022	I/II	Recruiting	NCT04180215
HPV-Specific T Cells	Baylor College of Medicine	United States	October 2022	I	Active, not recruiting	NCT02379520

*IL-12* interleukin-12; *Allovectin-7®* HLA-B7/β-2 microglobulin plasmid DNA/lipid complex; *E6 TCR E6* T cell receptor; *HPV* Human papilloma virus

## Challenges and perspective

The occurrence of malignant tumors, including oral cancers, results from the accumulation of genetic mutations and epigenetic modifications that lead to tumor-related phenotypes, including unlimited proliferation, apoptosis resistance, angiogenesis, invasion, and metastasis [195]. Moreover, in the process of cancer development, the tumor and the surrounding microenvironment constantly interact and evolve, forming characteristics conducive to tumor growth and invasion, leading to tumor development and metastasis [196]. For most patients, metastatic tumors remain an incurable disease that cannot be cured with traditional treatments. The development of tumor immunotherapy has brought hope for the cure of tumors, especially advanced tumors. Different from traditional therapeutic strategies, tumor immunotherapy mainly activates the host immune system and then mobilizes specific immune cells to recognize and kill tumor cells [197]. The immunotherapy can achieve a persistent anti-tumor response due to the host's immune memory effect. Therefore, immunotherapy can not only effectively inhibit the primary tumor but also further prevent tumor recurrence and inhibit metastasis, making it the only emerging therapy that is expected to completely cure tumors.

For decades, tumor immunotherapy has changed the treatment pattern of multiple solid tumors and hematological malignancies, bringing good news to tumor patients, especially patients with advanced malignant tumors or multidrug resistance. However, tumor immunotherapy is still faced with many difficulties, such as low immune response rate and lack of effective and reliable efficacy prediction markers, which is one of the biggest challenges to achieve accurate personalized immunotherapy. In addition, TME is a complex heterogeneous ecosystem, and the occurrence, development, and metastasis of tumor are closely related to the internal and external environment of tumor cells. It not only affects the growth and metabolism of tumor cells but also affects the structure, function, and metabolism of tumor tissues. The various microenvironmental characteristics, including hypoxic microenvironment, metabolic microenvironment, acidic microenvironment, and mechanical microenvironment, have attracted the attentions of scientists and clinicians. Combining drugs that modulate TME with standard therapies, such as traditional chemotherapy regimens with drugs that improve the acidic environment of tumors, promises exciting therapeutic results.

With the continuous emergence and innovation of new technologies and new methods, and the increasingly close interdisciplinary integration, tumor immunotherapy ushers in the rapid development, showing great potential to cure tumors. However, due to the heterogeneity of tumor and the difference of individual immune environment, immunotherapy cannot show good therapeutic effect in all individuals. The selection of specific targets, screening of suitable tumor patients, and the combined application of multiple therapies can partially solve the current problems of tumor immunotherapy. The development of immunotherapy drugs and related clinical trials based on multiple targets have provided a new direction for clinical work and are expected to greatly improve the current plight of malignant tumor treatment. We look forward to exciting clinical studies that are underway or will soon be conducted, but we clearly recognize that there are still some limitations and need to be improved in the current approach to immunotherapy drugs and rational application. The development of tumor immunotherapy drugs should be based on solid and scientific theories and guided by the real clinical needs. In the future, the development of basic medicine, tumor immunology, pharmacy, bioinformatics, and other disciplines will further promote the development of tumor immunotherapy. Expanding benefit groups and improving efficiency are the direction of future research. Tumor immunotherapy designed and developed based on new theories, new technologies, and new methods can safely and effectively enhance the immune system, destroy tumor cells, and finally achieve the goal of cure with limited toxicity. Immunotherapy has an important development prospect and is a potential method for future clinical treatment of malignant tumors.

## Conclusions

Oral cancer severely affects the appearance, eating and even endangers life. There are few safe and efficient clinical treatment strategies for oral cancer, which is mainly due the complex tumor immune microenvironment. Tumor immunotherapy is the specific killing of tumor cells by activating the host's own immune system, which is not only suitable for the treatment of primary tumor but also has a specific killing effect on metastatic tumor. Therefore, tumor immunotherapy based on TME demonstrates a potential prospect in clinical anti-tumor therapy. Most importantly, a healthy attitude and lifestyle remain the first element in the prevention and treatment of oral cancer.

## Data Availability

Not applicable.

## Abbreviations

ACI: Adoptive cellular immunotherapy
anti-HER2: Anti-human epidermal growth factor receptor 2
BMDCs: Bone marrow dendritic cells
CAFs: Cancer-associated fibroblasts
CTLs: Cancer-specific T cells
CEA: Carcinoembryonic antigen
Car-T: Chimeric antigen receptor T
CD3AK: CD3AcAb-activatived killer
CRISPRi: Clustered regularly interspaced short palindromic repeats interference
CIK: Cytokine-induced killer
CTLA-4: Cytotoxic T lymphocyte antigen-4
DCs: Dendritic cells
EGFR: Endothelial growth factor receptor
EMT: Epithelial mesenchymal transformation
ECM: Extracellular matrix
EXTL3: Exostosin-like glycosyltransferase 3
FGF: Fibroblast growth factor
FDA: Food and Drug Administration
GM-CSF: Granulocyte-macrophage colony-stimulating factor
HBGF-1: Heparin binding growth factor-1
HGF: Hepatocyte growth factor
HLA: Human leukocyte antigen
HPV: Human papillomavirus
HA: Hyaluronic acid
HIF: Hypoxia-inducible factor
IC: Immune checkpoint
ICB: Immune checkpoint blockade
ICIs: Immune checkpoint inhibitors
IDO: Indoleamine-2,3-dioxygenase
IFN-α: Interferon alfa
IFN-γ: Interferon γ
IGF: Insulin-like growth factor
IL-10: Interleukin-10
IL-12: Interleukin-12
LAG-3: Lymphocyte activation gene 3
LAK: Lymphokine-activated killer
mRNA: Messenger RNA
MHC: Major histocompatibility complex
MMPs: Matrix metalloproteinases
M-CSF1: Macrophage colony-stimulating factor 1
mAbs: Monoclonal antibodies
NCI: National Cancer Institute
OPVL: Oral proliferative verrucous leukoplakia
OSCC: Oral squamous cell carcinoma
PI3k: Phosphoinositide 3-kinase
PDGF: Platelet-derived growth factor
PD-1: Programmed cell death-1
PD-L1: Programmed cell death-ligand 1
Ras: Rat sarcoma
RGD: Arginine-glycine-asparagine
STAT3: Signal transducer and activator of transcription 3
TIM-3: T cell immunoglobulin-3
TRICOM: Triad of costimulatory molecules
TAA: Tumor-associated antigen
TAM: Tumor-associated macrophages
TGF-β: Transforming growth factor
Tregs: Regulatory T cells
TILs: Tumor-infiltrating lymphocytes
TNF-α: Tumor necrosis factor
TSA: Tumor-specific antigen
ROS: Reactive oxygen species
TME: Tumor microenvironment
VISTA: V-domain Ig suppressor of T cell activation
VEGF: Vascular endothelial growth factor
VEGFR: Vascular endothelial growth factor receptor
WHO: World Health Organization
